# Prevalence, predictors and outcome of carotid stenosis: a sub study in the Preventive Antibiotics in Stroke Study (PASS)

**DOI:** 10.1186/s12883-020-02032-4

**Published:** 2021-01-12

**Authors:** Twan J van Velzen, Laurien S Kuhrij, Willeke F Westendorp, Diederik van de Beek, Paul J Nederkoorn

**Affiliations:** Department of Neurology, Amsterdam UMC, location Meibergdreef, Meibergdreef 9, 1105 AZ Amsterdam, The Netherlands

**Keywords:** Carotid stenosis, Ischaemic stroke, Prevalence, Predictors, Survival

## Abstract

**Background:**

The prevalence of carotid artery stenosis (CAS) in acute ischaemic stroke (AIS) patients is historically reported at 15–20%, but an up-to-date estimate is lacking. We hypothesise it is lower than historically reported, due to better risk management to date. The study aims to study prevalence, predictors and survival of CAS in AIS patients.

**Methods:**

We included patients with AIS from the Preventive Antibiotics in Stroke Study (PASS), a large Dutch randomized, multicentre, open-label phase III trial that included 2538 patients with acute stroke and randomised between standard care or preventive ceftriaxone. Patients with stroke in the anterior circulation that underwent diagnostic testing of the internal carotid artery (ICA) were eligible for this sub study and used in these secondary analyses. Logistic regression analyses were performed to identify predictors for CAS ≥ 50%. Additionally, an ordinal regression was performed to assess the association between presence of CAS at baseline and functional outcome at three months on the modified Rankin scale (mRS).

**Results:**

1480 patients with AIS were included; 277 had CAS (18.7%; 95%CI:17.7-19.7). Age, hypertension, smoking and male gender were found as best-fit predictors for presence of CAS. Significant shift in mRS score after 90 days for CAS ≥50% towards a higher mRS score with an OR of 1.66 (95% CI 1.30-2.10) was found.

**Conclusions:**

Current prevalence of CAS is 18.7%, which is higher than we expected. Gender, smoking and hypertension are important factors associated with CAS. Patients with CAS had a significantly higher mRs score after 90 days.

**Trial registration:**

Unique identifier:ISRCTN66140176

## Background

The prevalence of internal carotid artery stenosis (CAS) of ≥ 50% in acute ischaemic stroke (AIS) patients has previously been estimated to be between 15% and 20% [1–3] . All of the reported patient populations were collected around the year 2000. Since then, the prevalence of several risk factors for cardiovascular disease (CVD) has decreased and treatment of risk factors effects has largely been improved [4]. Some of these factors are known predictors for carotid disease specifically, e.g. hypertension and smoking [5, 6]. In addition, statins are widely used to date. With the declining number of risk factors for carotid disease, we hypothesize that the prevalence of CAS has decreased. The prevalence of CAS is important as it indicates the burden of disease and the scale of patients that might benefit from changes made to the current protocol for treatment of CAS. Furthermore, it is an indication of the impact of changes that have been made in treatment of CVD and the healthier life style that is thought to have taken place in recent years. Finally, a valid estimate is needed for power calculations for novel studies. The aims of this study are to estimate the current prevalence of CAS in AIS patients, to identify predictors for CAS and describe survival in these patients.

## Methods

### Data source

Data from the Preventive Antibiotics in Stroke Study (PASS) cohort were used for these secondary analyses. PASS is a large, multicentre, randomised, open-label trial with masked endpoint assessment investigating the effect of preventive antibiotic therapy on functional outcome at three months in patients with AIS or haemorrhagic stroke with a National Institutes of Health Stroke Scale (NIHSS) score ≥ 1. Patients with infarction or occlusion in more than one vessel territory or patients with transient ischemic attack (TIA) are potentially included, since data on multi-vessel problems were lacking. Patients with infarction in the posterior circulation were excluded. The study was conducted between 2010 and 2014 and results were published in 2015 [7, 8].

### Inclusion criteria

For this sub study, patients with AIS with symptoms of the anterior cerebral circulation from the PASS cohort were included. Patients whose diagnostic test, performed after the ischemic stroke, results regarding the carotid artery stenosis or the possible predictive factors of CAS were unavailable were excluded. Computer tomography angiography (CTA), magnetic resonance angiography or Doppler ultrasound were used to determine the presence of CAS. The presence of CAS was defined as stenosis in the internal carotid artery ≥ 50%. The additional data on presence of CAS was retrospectively derived from the medical records, whereas al other baseline data in PASS were collected prospectively. Reports about the degree of stenosis of the carotid artery as assessed by a radiologist were used. For measuring degree of stenosis on CTA or MRA the NASCET criteria were used, if duplex ultrasound was performed a standardised ratio to determine percentage of stenosis was used, as recommended by the radiologists association. All medical reports were searched through manually retrospectively, either in the specific hospital or through online possibilities to get access to the patient files, in order to retrieve reports by the radiologists in the acute moment after the ischemic stroke. No imaging done before the stroke was used as predictor of CAS. Information regarding the possible predictive factors was derived from the PASS database and used in this sub study.

### Outcomes

The primary outcome is the prevalence of CAS ≥ 50%. Secondary outcomes are predictors of CAS ≥ 50% and the association between CAS ≥ 50% and functional outcome at 90 days after stroke, defined as the modified Rankin Scale (mRs) score < 3. This outcome was assessed by a structured telephone interview by trained trial nurses [7].

### Statistical analysis

Chi-square tests were used to test for differences in dichotomous variables. Mann-Whitney U test and Kruskal-Wallis tests were used to test for differences in age and NIHSS score/mRS respectively as these factors were not normally distributed. Consecutively, we performed univariate followed by multivariable logistic regression analysis to identify predictors for CAS ≥ 50%. If the number of missing variables exceeded 5%, this specific variable was excluded from the analysis. If the number of missing variables was below 5%, cases with a missing variable were not used in that specific analysis. The following possible predictors were tested, based on clinical grounds: atrial fibrillation (AF) or flutter, hypercholesterolemia, hypertension, myocardial infarction, peripheral vascular disease, obstructive pulmonary function or chronic obstructive pulmonary disease, and smoking (both current and former). The predictors with a p-value < 0.1 after univariate logistic regression or based on clinical relevance were included for the multivariate logistic regression analysis. We used backward selection to find the best-fit model and the model was tested for multi-collinearity with testing of the variance inflation factor. An ordinal regression was performed to analyze whether there is a significant shift in mRS after 90 days between patient with and without CAS. Age (rounded), NIHSS at baseline, pre-stroke mRS, gender and acute treatment (intravenously thrombolysis) were used as confounders. Age and NIHSS at baseline were used as continuous factor, the others as categorical factors. Pre-stroke mRS is used as confounder since patients that are functionally less independent (have an higher mRS), could have a lower probability of improvement after stroke. Finally, a dichotomized analysis on mRS after 90 days was performed, once with mRS 0–2 versus 3–6 and once with mRS 0–5 versus 6, corrected for the same confounders as the ordinal regression analysis. Dichotomization on mRS 2 and 3 is used since both limits are used in large clinical trials as maximum mRS as indication for good outcome [9]. IBM SPSS Statistics version 25 was used for statistical analyses.

## Results

**Fig. 1:**
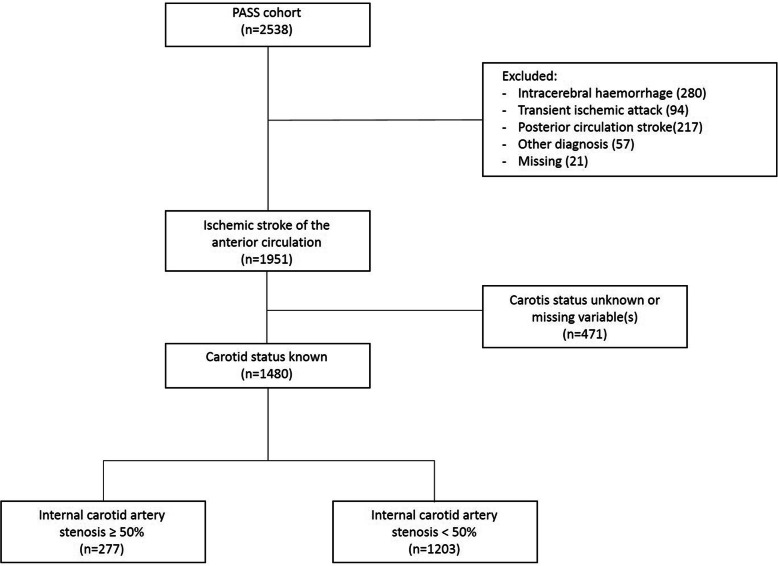
Patient selection flowchart

1951 patients with ischemic stroke in the anterior circulation were identified from the PASS cohort. Out of these patients, 471 patients (24.1%), no diagnostic test was performed or reported to assess the presence of CAS. (Fig. 1). In these patients AF was diagnosed more often than patients in which the diagnostic test for CAS was reported (26.7% versus 12.4%, *p* < 0.001). In the remaining 1480 patients, 277 had CAS (18.7%, 95%CI 17.7–19.7%).

### Patient characteristics

The patient characteristics are shown in Table 1. The mean age of patients with CAS was 72.5.

**Table 1:** Baseline characteristics

	ICA stenosis < 50% (*n* = 1203)	ICA stenosis ≥ 50% (*n* = 277)
Age (mean with standard deviation)	70.70 (± 12.5)	72.47 (± 10.8)
Hypertension	52.99%	61.01%
Hypercholesterolemia	26.7%	32.4%
Smoking	60.1%	70.3%
Gender (male)	56.8%	69.0%
NIHSS (median, IQR)	4 (3–7)	6 (4–12)
Thrombolysis	36.7%	43.3%
Pre-stroke modified Rankin Scale (median, IQR)	0 (0–1)	0 (0–1)
Modified Rankin Scale after 90 days (median, IQR)	2 (1–3)	2 (1–4)
Chronic obstructive pulmonary disease	6.5%	11.9%
Atrial fibrillation	12.6%	12.0%
Myocardial infarction	12.0%	16.6%
Peripheral vascular disease	7.1%	11.6%

(SD 10.8) and the median NIHSS was 6 (IQR 4–12). Most patients with a CAS suffered from hypertension (61%), had smoked prior to stroke (70%) and were male (69%). Patients with CAS received intravenous thrombolysis more frequent than patients without. Compared to the group without CAS, the median NIHSS is higher in the CAS group.

### Logistic regression analyses

Univariate logistic regression analyses showed several associations with the known predictors of presence of CAS (Table 2).

**Table 2:** Univariate logistic regression analyses with predictors of internal carotid artery (ICA) stenosis as independent variables and ICA stenosis ≥ 50% as the dependant variable

	*p*-value	Odds ratio (95% CI)
Age (year)	0.031	1.012 (1.001–1.023)
Hypertension	0.016	1.388 (1.063–1.812)
Hypercholesterolemia	0.060	1.312 (0.988–1.742)
Smoking	0.002	1.578 (1.182–2.107)
Gender (male)	< 0.001	1.691 (1.279–2.235)

Subsequently, a multivariable logistic regression was performed (Table 3).

**Table 3:** Multivariate logistic regression analyses with associated predictors of CAS as independent variables and CAS ≥ 50% as the dependant variable. Backward selection was used with the univariate associated variables

	*p*-value	Odds ratio (95% CI)
Age (year)	0.002	1.02 (1.007–1.033)
Hypertension	0.021	1.386 (1.05–1.83)
Smoking	< 0.001	1.782 (1.305–2.433)
Gender (male)	< 0.001	1.837 (1.38–2.447)

This analysis showed a best-fit model with age, hypertension, gender and smoking as variables for presence of CAS. The test for multi-collinearity indicated no multi-collinearity.

### Survival and functional outcome 90 days after stroke

**Fig. 2:**
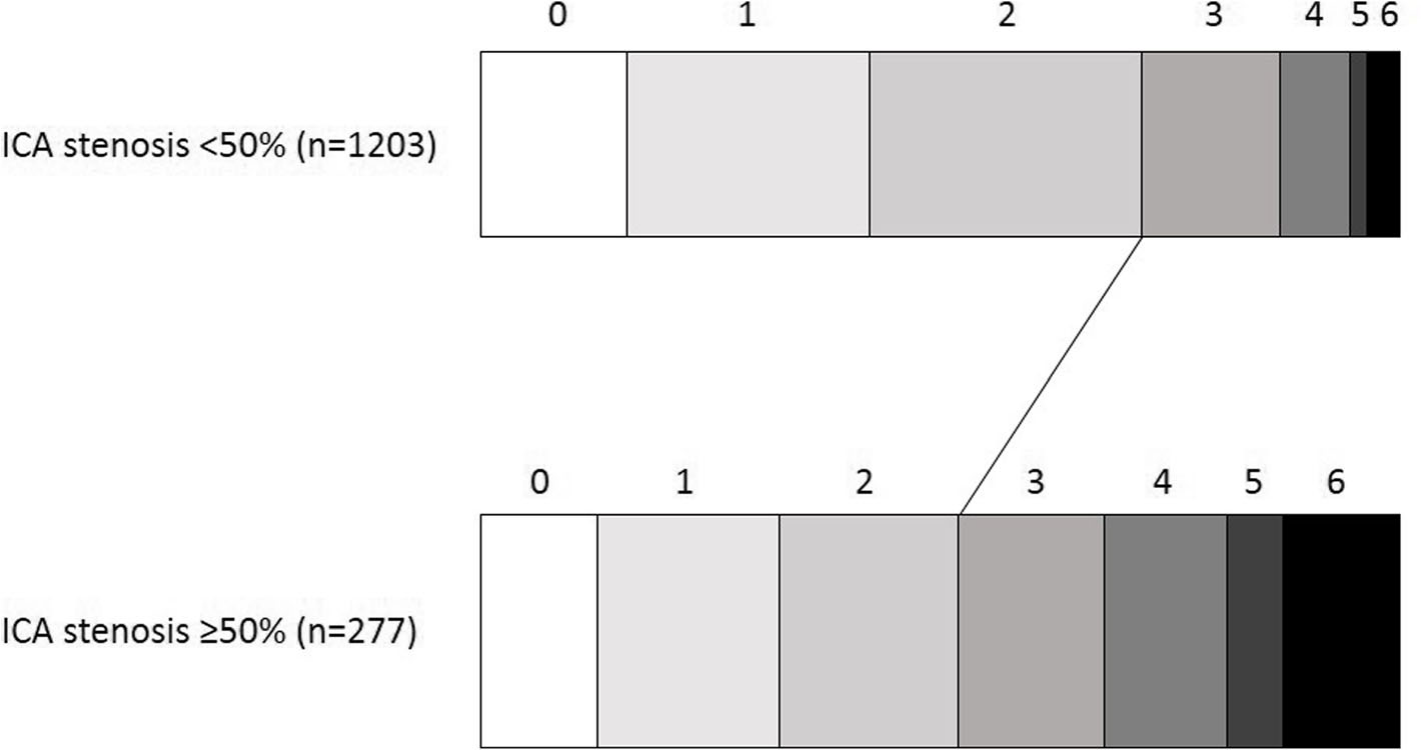
Shift in modified Rankin Scale (mRS) 90 days after stroke. Adjusted common odds ratio for CAS ≥ 50% of 1.66 (95% CI 1.30–2.10). The left side of the diagonal line (mRS 0–2) indicates a good functional outcome with a between groups difference of 20%. 0 = no remaining symptoms, 6 = dead

A median mRS after 90 days of 2 was found in both groups, with a different IQR (see Table 1). A multivariate ordinal regression analysis showed a significant shift towards a higher mRS in patients with CAS (Fig. 2) (*p* < 0.001). It showed an adjusted common odds ratio (OR) for CAS of 1.66 (95% CI 1.30–2.10), for male gender an OR of 0.76 (95% CI 0.63–0.91), for NIHSS of 1.18 (95% CI 1.16–1.21) and for thrombolysis an OR of 0.76 (95% CI 0.62–0.92). For pre-stroke mRS, only a significant shift was found for either mRS 0 and mRS 1, with OR for pre-stroke mRS of 0 of 0.29 (95% CI 0.16–0.52) and for pre-stroke mRS of 1 of 0.44 (95% CI 0.23–0.83). Age showed no significant relation with mRS (*p* = 0.078).

A significant difference was seen for mRS 0–2 versus > 2 between patients with a carotid stenosis compared to patients without, with a larger proportion of patients with a good functional outcome in the group without stenosis (72.0% versus 52.0%, *p* < 0.001). Mortality was significantly increased in patient with carotid stenosis with 12.6% in the stenosis group versus 3.5% in the group without stenosis (*p* < 0.001).

## Discussion

This sub study in PASS, a large Dutch stroke with acute stroke patients, showed an up-to-date prevalence of CAS in patients with AIS of 18.7% with age, hypertension, male gender and smoking identified as predicting factors for the presence of ICA stenosis. This prevalence is in line with published research, as well as the predictors for ICA stenosis that were found [2–6]. Ordinal regression analysis showed an adjusted common OR of 1.66 for ICA stenosis for a worse outcome after stroke in patients who also suffered from CAS. Dichotomized, the mRS showed a worse functional outcome in these patients as well, both in mortality and in less patients in the group with good functional outcome (mRS 0–2). An ICA stenosis ≥ 50% as a predictor for worse functional outcome after stroke is a new finding and has not been reported before.

Even though the prevalence that was found is in line with the previous reports on prevalence of CAS in AIS, it is higher than we expected. In 2007 Ford et al. reported that the decrease in deaths from coronary disease and they attributed 44% of the decrease to improvement of CVD risk factors. Mostly caused by the lowering of systolic blood pressure, reduction in percentage of smokers and lowering of blood cholesterol [10].  Furthermore, it has been reported that the overall diet in the population have become healthier and people have accustomed a less sedentary life-style [11–13]. Overall, CVD remain the most important cause of death worldwide. Even though western countries did see decreasing numbers of CVD death, with a sharper decrease in coronary death compared to stroke mortality, while developing countries saw an increase in both complications [14]. The fact that, in these recent data, we did not find a lower prevalence of CAS, as marker of large-vessel disease, remains surprising.

An important consideration is that the formation of carotid atherosclerosis and stroke as final effect take a substantial amount of time to develop. The patients that finally suffer from a stroke as a result of CAS are probably the patients with most comorbidities in the CAS group. The majority of patients with CAS that we included in this study, could have been established before the reduction of risk factors was started, as the formation of atherosclerosis takes multiple decades.

Another explanation could be that the decrease in risk factors is not of a sufficient significance or that other (unknown) risk factors become more important if you look at the overall effects after the reduction of the known risk factors. Taking these factors into account, one could hypothesize that it could take a longer time to find the decreasing prevalence of CAS caused by the measures decreasing CVD risk factors and CAS predictors.

The major strength of this study is the large number of AIS patients with complete follow-up that was used in this sub study. The most important limitation is that patients with TIA were excluded and that in a large number of patients (24.1% of the initial 1951 patients), it is unclear why the information regarding the status of CAS is lacking. This could cause an overestimation of the prevalence of CAS. A possible reason for missing this information is AF as cause of stroke. In these patients, the degree of stenosis of the carotid artery is often not investigated. Furthermore, octogenarians or patients with poor mRs or comorbidities, such as dementia, are often not eligible for revascularization and consequently are not investigated by imaging.

The worse functional outcome of patients with CAS is an interesting finding, even though PASS was not powered to show this difference, which deserves further attention. The general functioning of CAS patients and their overall high CVD risk could be an explanation for the worse outcome in these patients and thus making CAS a symptom of the worse outcome and not necessarily the cause of the worse outcome itself. Furthermore, CAS could be caused by the low-socio economic status that is correlated with most CVD risk factors that were found and are linked to worse outcome overall [15].

## Conclusions

We conclude that the prevalence of ICA stenosis remains as high as reported before and the same predicators were found. A shift towards worse outcome after 90 days has been found for CAS patients, but this needs further attention, for example by the following means. First, our findings demand further research on survival of patients with CAS, both symptomatic, as is done in this sub study, as in asymptomatic CAS. Second, these findings indicate the urgent need for stratified analysis of different causes of stroke, regarding outcome, survival and predictors of these patients. This could lead to better understanding of patient groups with worse outcome and a better prediction of stroke outcome. Finally, further decreasing smoking and increasing the treatment of hypertension in the population can potentially decrease the number of CAS in AIS.

## Data Availability

The datasets used and/or analysed during the current study are available from the corresponding author on reasonable request.

## Abbreviations

AIS: Acute ischemic stroke
AF: Atrial fibrillation
CAS: Carotid artery stenosis
CI: Confidence interval
CTA: Computed tomography angiography
CVD: Cardiovascular disease
ICA: Internal carotid artery
IQR: Interquartile range
mRS: Modified Rankin Scale
NIHSS: National Institutes of Health Stroke Scale
PASS: Preventive Antibiotics in Stroke Study
SD: Standard deviation
TIA: Transient ischemic attack
